# Ponseti Casting for Severe Club Foot Deformity: Are Clinical Outcomes Promising?

**DOI:** 10.1155/2015/821690

**Published:** 2015-02-10

**Authors:** Mohammad Hallaj-Moghaddam, Ali Moradi, Mohammad Hosein Ebrahimzadeh, Seyed Reza Habibzadeh Shojaie

**Affiliations:** ^1^Orthopedic Research Center, Mashhad University of Medical Science, Mashhad 91766-99199, Iran; ^2^Faculty of Medicine, Mashhad University of Medical Sciences, Mashhad, Iran

## Abstract

Between 2007 and 2010, a prospective study was done on 85 patients with severe idiopathic nonsyndromic clubfeet, in our center. Demographic features, severity of the deformity before and after serial casting according to Diméglio classification, and complications were assessed. The mean age of the patients was 8 days and 69% were male. The mean follow-up period was 26 months. The average number of castings used to correct the deformity was 5.7 times (range: 4 to 8). Tenotomy was performed in 76 (89.4%) of the feet. In all patients, plantigrade foot was achieved. Tenotomy occurred more in patients with higher Diméglio scores. Although patients who underwent Achilles tenotomy began to walk later than those who did not (13 ± 7.2 versus 9.2 ± 18), it was not significant (*P* = 0.06). Relapse rate, at the end of follow-up, was 27.1%. Diméglio score before casting was 16 ± 3.4 and at the end of follow-up it was 1.6 ± 6.2. The patients with bilateral clubfeet had inferior final outcome compared to those with unilateral clubfoot. Eighty percent of parents' were completely satisfied with their child's gait and foot appearance (94.1%). Ponseti method of manipulation and casting is a valuable technique in severe club foot as well as in common types.

## 1. Introduction

Idiopathic clubfoot is defined as isolated deformity of one or both limbs, which consists of four components: equines, hind foot varus, forefoot adductus, and cavus [1, 2]. Although clubfoot is one of the most known congenital deformities that occurs in one per 1000 live births [3], its management, especially in severe cases, is still challenging.

Etiologies such as vascular abnormalities [4], abnormal muscle insertion [5], and environmental factors such as in uteromalpositioning [6] were proposed to explain the occurrence of clubfoot deformity. Recently, it was proposed that genetic factors are more likely to be responsible [7–10]; however, it seems that clubfoot is considered a multifactorial disease [1].

Currently, closed management, such as serial casting is known as the gold standard treatment of idiopathic clubfoot [1]. Long-term studies have reported excellent results in using the Ponseti method of clubfoot manipulation and serial casting accompanied with Achilles tendon tenotomy and foot abduction brace [11–13]. Ponseti method has showed better range of motion and push-off strength compared to surgical release [14, 15]. Furthermore, extensive surgical release leads to more stiffness and arthritis in long-term studies [16].

In the present study, we have investigated how valuable the Ponseti method is in children with severe clubfoot deformities in our developing country. Moreover, we were curious to understand what factors lead to further relapse in severe clubfeet and the factors predicting tenotomy.

## 2. Material and Methods

In a prospective study, we reviewed clinical outcomes of 85 children with severe clubfeet who were admitted to the department of orthopedic surgery in our referral hospital between 2007 and 2011. We attempted to correct the clubfeet deformities with serial casting with Ponseti method, as was previously described [11]. Demographic features, severity of the deformity before and after serial casting, and complications were assessed. All patients' parents accepted and signed an informed consent. The Ethical Committee for Research of the Mashhad University of Medical Sciences approved the study.

### 2.1. Population

All patients had idiopathic nonsyndromic clubfeet. Only the patients with severe and very severe clubfeet deformity, according to the Diméglio classification [16], were enrolled into our study. The patients were younger than two months and none of them had previous surgical or nonsurgical treatment to correct the deformity. If both lower limbs were affected, we included only the more severe one into our study.

### 2.2. Data Collection

Two orthopedic surgeons with experience in the Ponseti method of manipulation and casting were involved in the treatment and follow-up of the patients. Before treatment, demographic data (age, sex, affected limb, familial history, and type of birth) were collected. During the follow-up period, frequency of castings, tenotomy procedure, complications related to tenotomy, and the time the child began to walk were recorded. Severity of deformity, before and after serial casting, was classified according to the Diméglio et al. classification: mild, moderate, severe, and very severe [16]. Parents' satisfaction was measured with two Likert questions (completely satisfied, quite satisfied, relatively satisfied, and not satisfied) indicating the shape and gait of the child after management. In the final visit, any alignment deviation (varus, valgus, adductus, and equines) of more than 10 degrees was considered significant.

### 2.3. Deep Sedation

We sedated the patients for tenotomy with mask and spontaneous ventilation, using 8% sevoflurane and 50% N_2_O-O_2_ induction. Later 4% sevoflurane and 1.5–2% isoflurane were used to end the procedure.

### 2.4. Casting Technique

We used the Ponseti method of manipulation and serial casting to correct deformity. Only tenotomy was applied under sedation in the operating room and other manipulations and castings were performed in a clinic. We manipulated each foot weekly to correct cavus, and we supinated the forefoot and externally rotated the foot. After each manipulation, we applied a long leg cast in the corrected position. The patients were observed closely for capillary filling and compartment syndrome a few hours later. We did not try to correct the equinus deformity before other malalignments had been corrected. While at least 70 degrees of foot external rotation was achieved, we decided between two choices of casting with or without Achilles tendon tenotomy. Percutaneous Achilles tenotomy under deep sedation in an operating room setting was preformed if there was any concern about further equinus (the foot could not be plantigrade). The last cast was worn for 3 to 4 weeks. When the serial casting came to an end, we managed the patients in a Dennis-Browne splint on a full-time basis for 6 months and subsequently on a part-time basis for 3 years. We did not use other braces or splints after completion of the treatment period with the Dennis-Brown splint. We followed the patients every 3 to 6 months during the first 2 years and every 6 to 12 months thereafter.

### 2.5. Statistical Evaluation

We used SPSS version 16 (SPSS Inc., Chicago IL) for descriptive and statistical analysis. For comparing two independent means of variables in subgroups, we used the independent* t*-test. Nonparametric variables were assessed with the Fisher Exact test and chi-square test. A *P* value of less than 0.05 was deemed to be significant.

## 3. Result

Eighty-five severe clubfeet underwent serial casting using the Ponseti method. The mean age of the patients at the time of first casting was 8 days (range: 1 to 60 days). Sixty-nine percent of all the clubfeet were male and 18% of the deformities were on the left side, while 21% were on the right and both limbs were affected in 61.2%. The mean age of the mothers was 25 years (range: 18 to 40 years). Sixty-one percent of patents were the firstborn child of the family. Forty-two patients (49%) were born with normal vaginal delivery and 43 of them (51%) were born with the caesarian method.

All patients were diagnosed at the time of delivery and the interval between diagnosis and treatment was 8.2 days (range: 1 to 60 days). The average number of castings that were used to correct the deformity was 5.7 times (range: 4 to 8) and all casts were changed with one-week intervals. At the last session, we decided to cut the Achilles tendon percutaneously in 76 (89%) of the cases. Manipulation and casting took a mean of 7.2 minutes for each procedure (range: 5 to 9 minutes) and 9.3 minutes in the last casting with the tenotomy under deep sedation (range: 6 to 11 minutes).

In all patients, plantigrade foot was achieved. The patients began to walk at a mean of 12 months (range: 8 to 18 months) and 83 patients (98%) could tolerate the Denis-Brown brace before starting to walk. Tenotomy was performed more in patients with higher Diméglio scores (16 ± 3.1 versus 14 ± 2.7, *P* = 0.01). Although patients who underwent Achilles tenotomy began to walk later than patients who did not (13 ± 7.2 versus 9.2 ± 18 months), but it was not significant (*P* = 0.06). More details are shown in Table 1.

The mean follow-up period was 26 months (range: 5 to 72 months). Relapse rate, at the end of the follow-up, was 27%. Diméglio score before casting was 16 ± 3.4 and at the end of follow-up it was 1.6 ± 6.2. More data is shown in Table 2.

The patients, who had both feet involved, had inferior final clinical outcomes comparing to those with unilateral clubfoot (2.1 ± 1.0 versus 0.63 ± 3.0, *P* = 0.03). Sex did not affect the outcomes (*P* = 0.16). Three patients had complications such as tenderness and pain at the site of the tenotomy (two cases) and mild infection (one) after Achilles tendon tenotomy. All of these three had very severe clubfeet. Parents' satisfaction regarding gait and appearance of the foot were complete in 80 (94.1%), quite in 4 (4.7%), relative 1 (1.2%), and no satisfaction in 0 (0%). Five parents had concerns about intoeing gait. All parents were completely satisfied with the foot shape except two: one because of metatarsal adductus and one for metatarsus adductus and heel varus. One patient had more than 15 degrees dorsiflexion, one had both residual heel adductus and equinus, and another had both heel adductus and metatarsal adductus. We could not find a relationship between relapse rate and other factors in severe clubfeet (Table 3). In multivariable analysis, the child age at the time of starting the serial casting and the initial severity of clubfoot deformity were two factors predicting the further tenotomy (Table 4).

## 4. Discussion

Treatment of severe clubfoot has always been a challenge. Extended surgical release leads to stiffness and further degenerative changes and it is not acceptable for the first step of treatment [17, 18]. Nowadays, manipulation and serial casting are the method of choice for clubfoot deformity [12, 13].

In our study, on average 5.7 serial casts were performed to correct the deformity. About 90% of patients needed a percutaneous Achilles tendon tenotomy. Also, the majority of the patients tolerated the Denis-Brown brace. Although it seems that patients with Achilles tenotomy began to walk later, it was not statistically significant. Most of the parents were satisfied with Ponseti method. Relapse occurred in 27% of clubfeet and the complication rate was not significant.

There were some limitations in our study. There was no control group to compare our results with other surgical and nonsurgical methods. Also, we did not do radiologic evaluations, so deformities were assessed only clinically.

We followed up the patients for 26 month, which is comparable with other studies assessing the Ponseti method [19–24]. The average age of our patients was 8 days. Other studies that were designed to follow up the patients at birth had the same average of 1 to 17 days [19, 21, 24]. Male to female patient ratio was similar to Panjavi et al.'s study [21], but it was higher than other studies such as Banskota et al. [20] or Pavone et al.'s studies [19]. In these two recent studies, all types of clubfeet were entered into the study, but our inclusion criteria were limited to severe cases. Bilateral clubfeet consisted of 62% of our patients, which is more than other studies [19, 20, 24, 25]. As our patients had severe clubfeet, it may explain the results.

In our study, the average number of castings was 5.7 times. It was more than Panjavi et al.'s study, which used general anesthesia for casting [21] but was less than other studies that used manipulation without sedation [19, 20]. More severe cases may lead to more casting in our study compared to Panjavi et al.'s [21].

Tenotomy rate was 90%, and other studies reported 79 to 93% [19, 21, 24]. Although patients with tenotomy started walking later, it was not significant. Niki et al. in a study evaluating the heeling of the Achilles tendon in infants [26] showed that the continuity of the tendon and its gliding were detected at four weeks, and the tendon continued to thicken up to three months and after one year it was similar to the opposite side. Marleix et al. in a systematic review advised Achilles tenotomy regardless of clubfoot severity [27].

Different studies used different braces to prevent further relapse. Sætersdal et al. compared two different unilateral and bilateral braces and concluded that compliance to all of the braces was good [24]. Orthosis noncompliance was noted from 6 to 23% in the literature [19, 21, 24]. Brace noncompliance is one of the most important causes of relapse [18, 20, 23]. In our study, brace compliance was as high as 97%.

Relapse rate was 8 to 23% in different studies [19–24], which was less than our results (28%). Our patients were severe and very severe, so it may explain the results [20]. Brace noncompliance [19, 21, 24], primary severity [21], below knee casting [27], and low level of parental education [28, 29] are the factors that increase failure rate.

Finally, it seems that in severe clubfeet, similar to usual cases, the Ponseti method of manipulation and casting are a valuable technique. Furthermore, the Achilles tenotomy is a procedure with low complication that does not seem to affect the child's walking.

## Figures and Tables

**Table 1:** Factors may relate to relapse in severe clubfeet managed with Ponseti method.

Variables	Normal clubfeet	Relapse clubfeet	Statistical test	*P* value
Sex (male/female)	42/18	17/8	Fisher's exact test	0.52
Age first casting started (day)	7.9	9	Independent *t*-test	0.56
Mother age (year)	25	26	Independent *t*-test	0.20
Accompanied with other deformities (yes/no)	3/57	2/23	Fisher's exact test	0.42
Age the child starts to walk (month)	13.0	12.2	Independent *t*-test	0.63
Number of castings	5.6	5.8	Independent *t*-test	0.77
Primary severity (Diméglio score)	15.90	16.01	Independent *t*-test	0.14
Tenotomy (yes/no)	54/6	22/3	Fisher's exact test	0.51
Satisfaction	1.2	1.2	Independent *t*-test	0.92

**Table 2:** Diméglio score, before serial casting and at final follow-up visit.

Classification	Before casting		At final visit	
	Cases	Frequencies	Cases	Frequencies
Plantigrade	62	73%	0	0
Mild	16	19%	0	0
Moderate	4	4.7%	0	0
Severe	2	2.4%	9	11%
Very severe	0	0	76	89%

**Table 3:** Factors may be associated with further tenotomy in clubfeet managed with Ponseti method.

Variables	With tenotomy	Without tenotomy	Statistical test	*P* value
Sex (male/female)	23/53	3/6	Fisher's exact test	0.98
Age at the time of first casting (day)	7.7	13	Independent *t*-test	0.06
Mother age (year)	25	26	Independent *t*-test	0.65
Accompanied with other deformities (yes/no)	3/74	2/6	Fisher's exact test	0.08
Number of castings	5.7	5.3	Independent *t*-test	0.22
Age the child starts to walk (month)	13	9.2	Independent *t*-test	0.06
Primary severity (Diméglio score)	16	14	Independent *t*-test	0.01
Relapse (yes/no)	22/54	3/6	Fisher's exact test	0.72
Final outcome (Diméglio score)	1.6	1.7	Independent *t*-test	0.97
Satisfaction	1.2	1.0	Independent *t*-test	0.62

**Table 4:** Multivariable analysis: factors may be associated with further tenotomy.

Variables	*B*	Standard error	*P* value	Odd ratio	95% Confidence interval for odd ratio	
Accompanied with other deformities	−4.96	1.50	0.071	0.01	0.0004	0.13
Age start walking (month)	−0.05	0.10	0.64	0.96	0.79	1.20
Age casting started	0.09	0.05	0.055	1.09	1.00	1.20
Primary severity (Diméglio)	−3.42	1.14	0.003	0.03	0.004	0.30
